# Tracking Blood Glucose and Predicting Prediabetes in Chinese Children and Adolescents: A Prospective Twin Study

**DOI:** 10.1371/journal.pone.0028573

**Published:** 2011-12-07

**Authors:** Guoying Wang, Lester Arguelles, Rong Liu, Shanchun Zhang, Wendy J. Brickman, Xiumei Hong, Hui-Ju Tsai, Binyan Wang, Houxun Xing, Zhiping Li, Xiping Xu, Xiaobin Wang

**Affiliations:** 1 The Mary Ann and J Milburn Smith Child Health Research Program, Children's Memorial Research Center, Chicago, Illinois, United States of America; 2 Division of Endocrinology, Department of Pediatrics, Northwestern University Feinberg School of Medicine and Children's Memorial Hospital, Chicago, Illinois, United States of America; 3 The Institute for Biomedicine, Anhui Medical University, Heifei, China; 4 Center for Population Genetics of Division of Epidemiology and Biostatistics, University of Illinois at Chicago, Chicago, Illinois, United States of America; 5 Division of Biostatistics and Bioinformatics, Institute of Population Health Sciences, National Health Research Institutes, Zhunan, Taiwan; National Institutes of Health - National Institute of Child Health and Human Development, United States of America

## Abstract

We examined the tracking of blood glucose, the development of prediabetes, and estimated their genetic contributions in a prospective, healthy, rural Chinese twin cohort. This report includes 1,766 subjects (998 males, 768 females) aged 6–21 years at baseline who completed a 6-year follow-up study. Oral glucose tolerance test was performed for all subjects at both baseline and follow-up. We found that subjects with low fasting plasma glucose (FPG) or 2 h post-load glucose (PG) levels at baseline tended to remain at the low level at follow-up. Subjects in the top tertile of baseline plasma glucose tended to have a higher risk of developing prediabetes at follow-up compared to the low tertile: in males, 37.6% vs. 27.6% for FPG and 37.2% vs. 25.7% for 2hPG, respectively; in females, 31.0% vs. 15.4% for FPG and 28.9% vs. 15.1% for 2 h PG, respectively. Genetic factors explained 43% and 41% of the variance of FPG, and 72% and 47% for impaired fasting glucose for males and females, respectively; environmental factors substantially contribute to 2hPG status and impaired glucose tolerance. In conclusion, in this cohort of healthy rural Chinese children and adolescents, we demonstrated that both FPG and 2hPG tracked well and was a strong predictor of prediabetes. The high proportion of children with top tertile of blood glucose progressed to prediabetes, and the incidence of prediabetes has a male predominance. Genetic factors play more important role in fasting than postload status, most of which was explained by unique environmental factors.

## Introduction

Prediabetes, defined as impaired fasting glucose (IFG) and/or impaired glucose tolerance (IGT), reflects intermediate levels of glucose dysregulation between euglycemia and diabetes and are fueling the epidemics of diabetes and cardiovascular disease worldwide [1]. Tirosh et al.[2] have shown that high normal fasting plasma glucose (FPG) levels, below 5.6 mmol/l, might be predictive of type 2 diabetes. In addition, recent study has indicated that the risk of cardiovascular morbidity and mortality is already significantly increased in those with modestly elevated FPG levels, even if their glucose levels are below the cutoff for diabetes [3], [4]. Thus, tracking plasma glucose from childhood through adolescence and into young adulthood may be helpful to identify the euglycemic children who are at risk for diabetes and cardiovascular disease.

Tracking is defined as the maintenance of a certain status (e.g., normal glucose tolerance or prediabetes) or a relative position within a distribution of values in a population over time [5]. Few studies have focused on the dynamics of blood glucose or on the effects of risk factors on the persistence of hyperglycemia from childhood to young adulthood. At present, there have been limited longitudinal studies in children and adolescents to identify baseline metabolic parameters associated with later development of prediabetes.

The ongoing longitudinal study of metabolic syndrome in Anqing, China offers a unique opportunity to address the above mentioned gaps. The objectives of this study were to examine the tracking of blood glucose and to identify predictors for the development of prediabetes in later life. A unique aspect of this study was the ability to estimate, using a twin design, the relative contribution of genetic and environmental factors to blood glucose and prediabetes. To our knowledge, this was the first longitudinal study of tracking both fasting and 2 h post-load (PG) in a large, healthy, predominantly rural Chinese population of children and adolescents.

## Methods

### Study population and Procedures

This report includes data from an ongoing study of metabolic syndrome in a large Chinese twin cohort. The population-based cohort of twin pairs was enrolled in Anqing, China from 1998 to 2000 (baseline) and then resurveyed in 2005–2006 (follow-up). Twins were chosen for the baseline survey based on the following criteria: (1) older than 6 years; (2) both twins available and willing to participate. Eligible twins were invited to a central office to complete a questionnaire interview, physical examination, and oral glucose tolerance test (OGTT). After a period of six years, eligible twins who met the following criteria: (1) both twins participated in the baseline survey; (2) both twins agreed and consented to participate in the follow-up study were invited to complete the follow-up study. During the time frame of the present study, 3837 children and adolescents were examined: 8 of these were excluded from the present analyses because they were diagnosed as diabetes at baseline or follow-up, 176 were excluded because data were missing for one of the predictor variables, and 1887 were excluded because they did not have follow-up examination due to immigrate to cities. Those without follow-up were older and more likely to be male, but, with respect to BMI, glucose, and insulin, individuals with follow-up were similar to the characteristics of the subjects which did not participate in the follow-up survey (data not shown). Therefore, participants selected for the present study consisted of 1,766 subjects (998 males, 768 females) free from diabetes, 6–21 years of age at baseline and 12-28 years at follow-up.

### Ethics Statement

The study protocol was approved by the Institutional Review Boards of Children's Memorial Hospital and the Biomedical Institute, Anhui Medical University in Hefei, China. Participants aged 18 years or older gave written informed consent; for participants younger than 18 years, written informed consent was obtained from a parent/guardian and the participant.

### Physical Activity

Physical activity was only obtained at follow-up visit using the short version of the international physical activity questionnaire (IPAQ-Short) (http://www.ipaq.ki.se). The “last 7 days” was used as the reference period, three types of physical activity information (vigorous, moderate and walking) were provided by participants across various physical activity domains (i.e., leisure time, occupational or school, domestic, and transport). Using both the total volume of activity and the number of activity days/sessions per week, the IPAQ generates a categorical indicator (low, moderate, and high) of regular physical activity.

### Anthropomorphic measures

Body weight was measured to the nearest 0.1 kg on a scale, and standing height to the nearest 0.1 cm with a stadiometer. For all measurements, the subjects were wearing only light indoor clothing and no shoes. Body mass index (BMI) was calculated as weight (kg) divided by height squared (m^2^).

### Laboratory assays

After a 12-hour overnight fast, each subject was performed a standard OGTT (1.75 g/kg or a maximum of 75 g of glucose). For glucose and insulin measurements, blood samples were drawn at two time points, e.g., 0 and 120 minutes after glucose administration. Plasma glucose was measured by a glucose oxidase enzymatic method using 7020 Automatic Analyzer (Hitachi, Tokyo, Japan). Standard samples that came with the reagents were used to perform standard quality control test. Plasma insulin was examined by electro-chemi-luminescence (ECL) method on an Elecsys 2010 system (Roche, Basel, Switzerland). Duplicate analyses were conducted in each day with coefficients of variation <10%. Insulin resistance was estimated using the homeostatic model assessment for insulin resistance index (HOMA-IR) which was calculated as fasting insulin concentration (µU/ml)×fasting glucose concentration (mmol/l)/22.5 [6]. Zygosity of each twin was determined using DNA fingerprint technology or microsatellite probes, as described previously [7].

### The definition for prediabetes and diabetes

The definition for IFG, IGT, and diabetes was based on the criteria of American Diabetes Association [8], [9]. A subject had IFG if FPG is between 5.6–6.9 mmol/L with 2hPG in OGTT of <7.8 mmol/l, and IGT as 2hPG in OGTT of 7.8–11.0 mmol/l with FPG <5.6 mmol/l. Prediabetes was defined as FPG between 5.6–6.9 mmol/L and/or 2 h value in OGTT of 7.8–11.0 mmol/L. A child or adolescent is considered to have a diabetes if his or her FPG is ≥7.0 mmol/l or 2 h PG is ≥11.1 mmol/l.

### Statistical analyses

All analyses were conducted using SAS version 9.1 (SAS Institute, Cary. NC). The distribution of HOMA-IR was positively skewed and a logarithmic transformation was used to normalize the data for subsequent statistical analyses. The persistence of plasma glucose over time was first examined graphically by constructing baseline age- and gender-specific tertiles of plasma glucose and then using a locally-weighted nonparametric smoothing function. Multiple linear regression models were used to examine baseline age- and gender-specific tertiles of FPG, 2hPG and HOMA-IR as independent variables in relation to each of these variables at follow-up; logistic regression was applied to examine effect of baseline variables on the risk of prediabetes at follow-up. All regression models were adjusted for age, Tanner stage, physical activity, smoking status and BMI. Generalized estimating equations (GEE) were applied to all regression models to adjust for intra-twin pair correlation and test the difference between baseline and follow-up.

Classical twin modeling approaches are performed on a comparison between the phenotypic correlation within MZ twins and also within DZ twins. Since MZ twins are genetically identical and DZ twins share on average 50% of the genes, a higher phenotypic correlation within MZ twins relative to DZ twins suggests a genetic component to the phenotype. An extension of this idea under a latent liability threshold model is structural equation modeling, which partitions the phenotypic correlations into what are known as ‘latent’ factors, the relative importance of which can be inferred by comparing the observed correlations (or concordances) between twins with predicted correlations (or concordances) if different sources of genetic and environmental factors were to play a role. In our model, we assume that additive genetics or the sum of effects of the individual alleles (a^2^) are the principal sources of variation—put another way, the heritability is the proportion of the phenotypic variance explained by additive genes. Other sources of variation that we assume in the model include common or shared environment (c^2^) and unique environment (e^2^) components. This approach uses a maximum-likelihood variance component method implemented in the statistical package Mx [10]. Script was downloaded from the GenomEUtwin Mx-script library (http://www.psy.vu.nl/mxbib). The results were obtained for the ACE models that allow for combinations of effects: A, additive effects of genes on multiple loci; C, environmental effects shared by twins in the same family; and E, environmental effects unique to the individual [11].

## Results

### Longitudinal changes of anthropometric and metabolic parameters

Baseline and follow-up anthropometric and metabolic parameters are presented by gender (Table 1). As expected, compared to the baseline variables, a significant increase at follow-up was observed for both genders, including height, weight, BMI, FPG, 2hPG, fasting plasma insulin (FPI), and HOMA-IR. Prevalence of prediabetes was higher in follow-up than baseline (p<0.001).

**Table 1:** General characteristics of 998 males and 768 females at baseline and follow-up*.

Variables	Males		(998)	Females	(768)	
	Baseline	Follow-up		Baseline		Follow-up
Age†	10.9±3.1	17.7±3.2		10.6±2.8		17.4±2.9
Height (cm)†	131.2±15.5	160.9±7.9		129.3±13.2		152.5±5.4
Weight (kg)†	28.2±10.8	49.9±9.0		26.8±9.0		46.5±6.6
BMI(kg/m^2^)†	15.8±2.4	19.2±2.5		15.6±2.3		19.9±2.4
FPG(mmol/l)†	4.5±0.5	5.4±0.4		4.4±0.5		5.3±0.4
2 h PG(mmol/l)†	4.7±0.9	5.8±1.4		4.7±0.8		5.9±1.3
FPI(µU/ml)†	7.1±5.7	8.2±4.1		7.5±6.4		10.7±4.8
HOMA-IR†	1.4±1.2	2.0±1.1		1.5±1.5		2.5±1.2
Prevalence†						
IFG	29(2.9)	281(28.2)		28(3.7)		145(18.9)
IGT	7(0.7)	75(7.5)		2(0.3)		50(6.5)
prediabetes	36(3.6)	320(32.1)		29(3.8)		175(22.8)
Physical activity						
low	-	272(27.3)		-		278(36.2)
moderate	-	312(31.3)		-		228(29.7)
high	-	263(26.4)		-		155(20.2)
N/A	-	151(15.1)		-		107(14.0)

*Data are presented as mean±SD or N(%); BMI, body mass index; FPG, fasting plasma glucose; 2hPG, 2 h post-load plasma glucose; FPI, fasting plasma insulin; HOMA-IR, homeostasis modeling assessment for insulin resistance index; IFG, impaired fasting glucose; IGT, impaired glucose tolerance; N/A, not applicable; †p<0.001 compared with baseline variables in males and females, separately.

### Tracking of FPG and 2hPG

**Figure 1:**
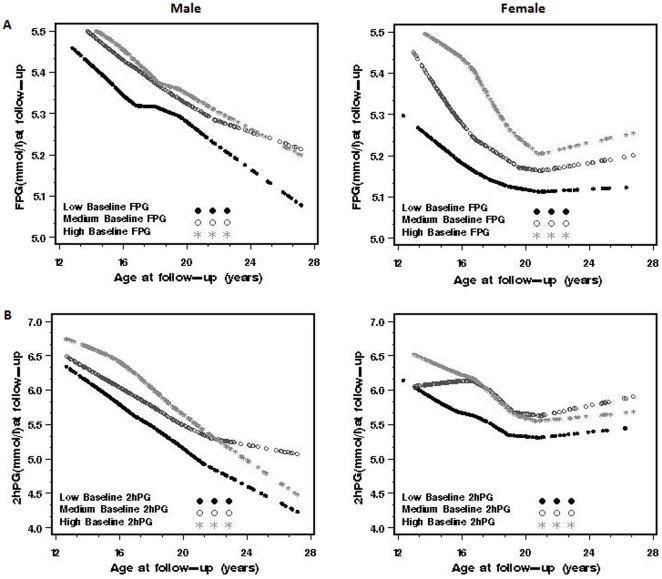
Plasma glucose levels at follow-up across age stratified by corresponding baseline levels. (A) Fasting plasma glucose at follow-up stratified by baseline FPG tertiles (low, middle and high) by gender. (B) 2 h post-load glucose at follow-up stratified by 2h PG tertiles at baseline by gender. All tertiles were ranked age- and sex-specifically. FPG, fasting plasma glucose; 2hPG, 2h post-load glucose.

In Figure 1, we clustered subjects into tertiles based on their baseline FPG and 2hPG levels, respectively. Next, we examined the tracking of FPG and 2hPG at follow-up. For both genders, subjects with low FPG and 2hPG levels at baseline tended to remain at the low level at follow-up. Likewise, individuals with middle or high FPG and 2hPG levels at baseline were more likely to remain at a middle or high level at follow-up. For example, 67.3% and 76.0% of individuals who ranked middle or top tertile with respect to FPG at baseline also did so at follow-up in males and females, respectively; for 2hPG 74.3% and 73.1% of subjects remained at middle or top tertile from baseline to follow-up in males and female, respectively. In general, FPG tracked stronger in females than males (p = 0.021), but no difference in 2hPG tracking between both genders.

### Correlations between baseline and follow-up parameters

We performed gender-stratified multiple linear regression analysis after adjusting for age, BMI, Tanner stage, smoking and physical activity to determine the predictability of selected baseline variables such as FPG, 2hPG, and HOMA-IR at follow-up. As shown in Table 2, after adjustment for above confounders, FPG and 2hPG at baseline were associated with FPG and 2hPG at follow-up in both sexes. For example, in males, relative to T1 FPG and 2hPG, both T3 FPG and 2hPG were associated with a 0.10 higher FPG at follow-up. Likewise, FPG at follow-up was higher for T2-T3 FPG and 2hPG than for each T1 glucose measure at baseline in females. For 2hPG at follow-up, T2-T3 2hPG at baseline increased 0.29 and 0.51 than T1 in males; an association with 0.39 and 0.42 higher 2 h PG was seen for T2-T3 in females. For FPG at follow-up, the most explanatory predictor is FPG (β = 0.10 and 0.21 in males and females, respectively); for 2hPG, the most explanatory predictor is 2hPG (β = 0.51 and 0.42, respectively); and the best predictors of follow-up HOMA-IR were the baseline levels of FPG in both genders. Moreover, the results were not changed by the inclusion of additional adjustment of percentage body fat (data not shown).

**Table 2:** Associations between baseline and follow-up plasma glucose and HOMA-IR.

Baseline	Follow-up variables*							
variables	FPG(mmol/l)		2h PG(mmol/l)		Log(HOMA-IR)		Prediabetes	
	Mean ± SD	β(SE)	Mean ± SD	β(SE)	Mean ± SD	β(SE)	OR	95%CI
**Males**								
**FPG**								
T1(low)	5.32±0.38	Ref	5.73±1.43	Ref	0.53±0.55	Ref	1.00	
T2	5.39±0.36	0.07(0.03)‡	5.85±1.32	0.15(0.10)	0.55±0.53	0.03(0.04)	1.23	0.87-1.73
T3(high)	5.41±0.39	0.10(0.03)‡	5.90±1.30	0.19(0.10)	0.60±0.49	0.08(0.04)†	1.66	1.18-2.33‡
P _trend_		0.0010		0.0548		0.0764		0.0032
**2h PG**								
T1(low)	5.31±0.39	Ref	5.53±1.30	Ref	0.55±0.49	Ref	1.00	
T2	5.39±0.36	0.08(0.03)‡	5.84±1.28	0.29(0.10)‡	0.55±0.49	0.02(0.04)	1.42	1.00-2.01†
T3(high)	5.41±0.38	0.10(0.03)‡	6.09±1.41	0.51(0.10)§	0.57±0.58	0.03(0.04)	1.71	1.21-2.41‡
P _trend_		0.0011		<0.0001		0.4668		0.0024
**HOMA-IR**								
T1(low)	5.37±0.37	Ref	5.80±1.40	Ref	0.54±0.54	Ref	1.00	
T2	5.38±0.39	0.02(0.03)	5.88±1.37	0.08(0.10)	0.55±0.53	0.02(0.04)	1.27	0.91-1.78
T3(high)	5.37±0.38	0.01(0.03)	5.79±1.27	-0.03(0.10)	0.59±0.50	0.04(0.04)	1.03	0.73-1.45
P _trend_		0.8629		0.7713		0.2916		0.8616
**Females**								
**FPG**								
T1(low)	5.16±0.36	Ref	5.83±1.26	Ref	0.74±0.46	Ref	1.00	
T2	5.25±0.36	0.09(0.04)‡	5.82±1.22	-0.01(0.11)	0.82±0.44	0.07(0.04)	1.61	1.01-2.55†
T3(high)	5.37±0.45	0.21(0.04)§	5.94±1.32	0.07(0.11)	0.90±0.48	0.13(0.04)§	2.56	1.64-3.99§
P _trend_		<0.0001		0.5345		0.0030		0.0000
**2h PG**								
T1(low)	5.19±0.38		5.58±1.23		0.81±0.46		1.00	
T2	5.28±0.42	0.09(0.04)†	5.97±1.25	0.39(0.11)§	0.83±0.46	0.03(0.04)	1.82	1.16-2.88‡
T3(high)	5.31±0.43	0.12(0.04)‡	6.03±1.26	0.42(0.11)§	0.82±0.47	0.01(0.04)	2.20	1.40-3.45§
P _trend_		0.0013		0.0001		0.7745		0.0007
**HOMA-IR**								
T1(low)	5.23±0.40	Ref	5.84±1.34	Ref	0.76±0.45	Ref	1.00	
T2	5.27±0.41	0.06(0.04)	5.94±1.31	0.09(0.11)	0.83±0.48	0.06(0.04	1.12	0.73-1.71
T3(high)	5.29±0.43	0.07(0.04)	5.81±1.14	-0.06(0.11)	0.87±0.46	0.09(0.04)†	1.01	0.66-1.57
P _trend_		0.0575		0.6095		0.0129		0.9480

*FPG, fasting plasma glucose; 2h PG, 2 h post-load plasma glucose; HOMA-IR, homeostasis modeling assessment for insulin resistance index; T, tertile.

†p<0.05,

‡p<0.01,

§p<0.001; adjusted for age, tanner stage, smoking, BMI, and physical activity at follow-up.

### Predictors for the development of prediabetes at follow-up

**Figure 2:**
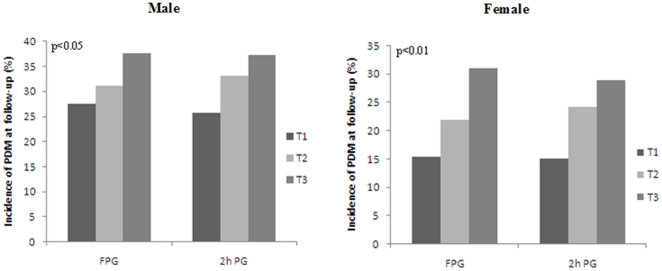
Association between baseline plasma glucose tertiles and incidence of prediabetes at the 6-year follow-up. All subjects had normal glucose tolerance at baseline (n = 1699). Subjects who ranked in the higher age- and sex-specific tertile of baseline fasting and 2h post-load plasma glucose developed more prediabetes when they were 12 to 27 years old. PDM, prediabetes at follow-up; T, tertile of blood glucose at baseline. P<0.05 and p<0.01 compared with T1.

We further examined the incidence of prediabetes at follow-up. A total of 27.6% (32.0% in males and 21.8% in females) of 1,701 normal glucose tolerance (NGT) subjects at baseline developed prediabetes at follow-up. Males have higher prediabetes than females at follow-up (p<0.0001). Furthermore, we grouped subjects into tertiles based on baseline plasma glucose. Subjects in the top tertile of baseline plasma glucose tended to have a higher risk of developing prediabetes at follow-up compared to the low tertile (in males, 37.6% vs. 27.6% for FPG and 37.2% vs. 25.7% for 2hPG, respectively; in females, 31.0% vs. 15.4% for FPG and 28.9% vs. 15.1% for 2h PG, respectively) (Figure 2).

### Heritability estimation for blood glucose, HOMA-IR, and prediabetes

Furthermore, we evaluated the genetic and environmental contributions to FPG, 2hPG, HOMA-IR, IFG and IGT at follow-up. The univariate structural equation modeling suggested that the ACE model was the best-fitted model, with genetic factors explaining 0.43 (95%CI: 0.19–0.70), 0.27 (95%CI: 0.00–0.55), 0.57 (95%CI: 0.34–0.66), 0.72 (95%CI: 0.18–0.83) and 0.27 (95%CI: 0.00–0.87) of the variance in FPG, 2hPG, HOMA-IR, IFG and IGT, respectively, in males. In females, genetic factors explained 0.41 (95%CI: 0.13–0.74), 0.13 (95%CI: 0.00–0.52), 0.49 (95%C: 0.11–0.64), 0.47 (95%CI: 0.00–0.86) and 0.39 (95%CI: 0.00–0.71) of the variance in FPG, 2hPG, HOMA-IR, IFG and IGT, respectively (Table 3). Generally, heritability estimations of FPG, 2hPG, HOMA-IR and IFG were higher in males than females, except for IGT, which was higher in females than in males. Unique environmental factors explained half of variance for 2hPG and IGT in both genders, except IGT in male which was explained by environmental factors in common.

**Table 3:** Heritability estimates for blood glucose, insulin resistance index and prediabetes*.

Variables	Correlation coefficients		Parameter Estimates		
	MZ	DZ	a^2^ (95%CI)	c^2^ (95%CI)	e^2^ (95%CI)
**Males**	**218(pairs)**	**141(pairs)**			
FPG	0.63	0.42	0.43(0.19∼0.70)	0.19(0.00∼0.43)	0.36(0.29∼0.44)
2hPG	0.57	0.45	0.27(0.00∼0.55)	0.33(0.07∼0.55)	0.40(0.32∼0.49)
HOMA-IR	0.58	0.18	0.57(0.34∼0.66)	0.00(0.00∼0.20)	0.43(0.34∼0.54)
IFG	66.7	43.9	0.72 (0.18∼0.83)	0.00 (0.00∼0.48)	0.28 (0.17∼0.43)
IGT	43.8	40.0	0.27 (0.00∼0.87)	0.45 (0.00∼0.80)	0.28 (0.11∼0.53)
**Females**	**201(pairs)**	**81(pairs)**			
FPG	0.71	0.50	0.41(0.13∼0.74)	0.31(0.00∼0.56)	0.29(0.23∼0.36)
2hPG	0.47	0.30	0.13(0.00∼0.52)	0.32(0.00∼0.52)	0.55(0.46∼0.66)
HOMA-IR	0.55	0.31	0.49(0.11∼0.64)	0.07(0.00∼0.41)	0.44(0.36∼0.54)
IFG	60.2	38.5	0.47 (0.00∼0.86)	0.27 (0.00∼0.79)	0.26 (0.14∼0.44)
IGT	23.1	0.00	0.39 (0.00∼0.71)	0.00 (0.00∼0.62)	0.61 (0.29∼1.00)

*FPG, fasting plasma glucose; 2hPG, 2h post-load plasma glucose; HOMA-IR, the homeostatic model assessment for insulin resistance; IFG, impaired fasting glucose; IGT, impaired glucose tolerance; MZ, monozygotic; DZ, dizygotic; a^2^, additive effects of genes on multiple loci; c^2^, environmental effects shared by twins in the same family; e^2^, environmental effects unique to the individual.

## Discussion

Our longitudinal study of 1,766 subjects represents the largest study to date of gender-specific tracking patterns of both FPG and 2hPG. This report contributes new information to our understanding regarding the dynamic change of plasma glucose from children to adulthood. First of all, our study demonstrated that both FPG and 2hPG tracked well; higher FPG or 2hPG levels within the normoglycemic range constituted an independent risk factor for future recognizable normal but higher blood glucose concentration and prediabetes. Especially, 2hPG can predict not only 2hPG, but also FPG in future. Second, it showed genetic factors contribute more to fasting than post-load status. Finally, our data document prevalent prediabetes incidence rate even in this relatively lean, healthy rural Chinese population and only over 6 years, and the incidence of prediabetes has a male predominance.

In general, the risk of progression from NGT to prediabetes in this lean population was relatively high, with total 27.6% of subjects progressed during the 6-year follow-up. It is consistent with the study in U.S. population [12], in which IFG prevalence among U.S. adolescents in 2005–2006 was 87.1% higher than the estimate from 1999–2000. The prevalence of prediabetes at baseline is much lower in this population compared to that the prevalence in children of a similar age in the United States [12]. We also estimate that the prevalence of IFG is 1.5 time lower among the adolescents age 12–19 years in our cohort compared to adolescents of a similar age in the US [13]. The dramatic increase in the prevalence of prediabetes after a 6-y interval may be attributable to the following reasons: (1) at follow-up, most of the subjects were at puberty, a period of increased insulin resistance [14]; (2) twins are more insulin resistant than singletons[15]; (3) rapid economic, nutritional, and lifestyle changes observed in China over the recent decades. In particular, diet changes that includes a greater consumption in fat and sugar or refined carbohydrates and less traditional food consumption (rice and vegetables) likely a direct result of the rapid economic growth and might explain the observed changes in prevalence of prediabetes.

In agreement with other study [12], males are at a relatively higher risk of prediabetes than females. This underlying mechanism is not yet understood. Central adiposity has been implicated as a risk factor for insulin resistance and type 2 diabetes[16] , and puberty is marked by rapid changes in body size, shape, and composition. Although girls had higher total body fat percentage during puberty, boys increased more central obesity [17]. Adiponectin has also been implicated as a risk factor for prediabetes, and a study has shown that serum adiponection levels decreased significantly at mid puberty in males, but values did not change during puberty in girls[18]. These differences in gender may be one explanation that FPG tracked better than males, however the exact mechanism behind the relationship of central obesity and adiponectin level and prediabetes and diabetes need to be further examined.

Several studies in adults have shown that blood glucose predicts subsequent diabetes and prediabetes [2], [19]. We found an increased risk of prediabetes in later life across tertiles of FPG and 2hPG levels within the normal range at baseline; this increase was independent of age, Tanner stage, smoking, BMI and physical activity. It suggests that elevated normal both FPG and 2hPG levels in childhood may predict risk of prediabetes in adulthood. Our findings with regard to prediabetes risk are consistent with findings from other previous studies [2], [20]. These cross-sectional studies found that high normal range blood glucose and adverse changes in glucose were significant influences on the risk of prediabetes and diabetes in young adolescents. Our findings extend previous report that baseline FPG itself was an independent predictor of diabetes [21]. In contrast with Nguyen et al report [21], HOMR-IR was not a good predictor for FPG, 2hPG and prediabetes in later life in our study population. A potential explain for discrepancy may be that our study samples are relative lean and with low plasma insulin concentration. Our data suggest that FPG or 2hPG, not HOMA-IR is the best predictor of prediabetes, potentially diabetes in relative lean population. Because prediabetes is an intermediate and reversible stage in the development of type 2 diabetes, early detection and appropriated management of high blood glucose in normoglycemic range among children and adolescents could effectively prevent or delay their development of type 2 diabetes in later life. The importance of earlier plasma glucose elevation and its persistence into adulthood is underscored by the fact that complications related to diabetes, such as diabetic nephropathy and retinopathy, are associated with duration of diabetes. Accordingly, it is important to develop a screening tool to identify high risk individuals and then suggest lifestyle modifications to prevent or delay the development of diabetes. In keeping with the findings from a previous study[19], we observed that subjects with higher FPG and 2hPG levels but in a normal range at childhood, compared with subjects who had a lower FPG or 2hPG level at childhood, were at a higher risk for glucose metabolism deterioration. Our findings are consistent with previous observation that elevated FPG levels within the normoglycemic range can predict diabetes [2], [22]. A novel finding is that 2hPG at childhood also is a best predictor of prediabetes at adulthood. Thus, the persistence of elevated blood glucose may assist clinicians in identifying individuals in need of targeted diabetes screening earlier.

It is well known that diabetes is generally considered a multifactorial disorder resulting from the interaction of genetics and environmental factors. Studies of twin pairs offer a powerful method of partitioning shared genetic, shared environmental and unique environmental sources of covariance of quantitative traits that are potentially relevant to the development of diabetes or prediabetes. We found that genetic factors may explain half of the variance in FPG and IFG, indicating that genetic factors play an important role in determining blood glucose and prediabetes. It confirmed the concept that a family history of diabetes was associated with elevated FPG levels [23]. In consistent with previous study [24], our data also showed that the genetic estimation for post-load status was lower than it was for fasting. This implies that genetic factors explain more of the variance in fasting traits related to glucose metabolism than do post-load status; whereas, 2hPG and IGT were predominantly related to unique environmental factors. This information suggests that individuals with elevated normal 2hPG, but without a family history of diabetes had better to take OGTT to diagnose IGT and diabetes, and may benefit from lifestyle changes. On the other hand, FPG screening for individuals with a family history of diabetes is more cost-efficient.

This study had several strengths. First of all, the data were generated from a rural population-based twin cohort with accurate ascertainment of zygosity and physical activity. Second, study participants were healthy, non-obese rural Chinese children and adolescents at baseline and who completed a six-year follow-up study. This population is less likely confounded by medication use and sedentary lifestyles. Third, the relatively homogeneous environment to which participants in our study were exposed might have reduced the effect of other unknown confounders.

Our study also has limitations. First, in our current study, we did not account for family history of diabetes due to inaccuracies related to self-reported family history in this rural population in which individuals could not be adequately tested for plasma glucose to obtain a diagnosis of diabetes. Moreover, OGTT is not always reproducible, but we only have one OGTT for each individual at each time point. Finally, diet plays an important role in the risk of prediabetes, but a limitation of this study was the lack in diet information.

In summary, in this cohort of healthy rural children and adolescents, we demonstrated that both FPG and 2hPG tracked well over 6 years and were strong predictors of the development of prediabetes. A high progressing rate to prediabetes was occurred in this twin population. Genetic factors substantially contribute to fasting glucose concentration and IFG; whereas, most of the variance in post-load glucose was influenced by unique environmental factors. Our findings underscore that the increase of blood glucose during growth and development at childhood should be care seriously. A simple FPG screening in children and adolescents may not only be practical but also critical to help identify individuals at high risk for the development of prediabetes and diabetes.
